# Interlayer Structure Engineering of MXene‐Based Capacitor‐Type Electrode for Hybrid Micro‐Supercapacitor toward Battery‐Level Energy Density

**DOI:** 10.1002/advs.202100775

**Published:** 2021-06-17

**Authors:** Wenxiang Cheng, Jimin Fu, Haibo Hu, Derek Ho

**Affiliations:** ^1^ School of Physics and Materials Science Key Laboratory of Structure and Functional Regulation of Hybrid Materials Ministry of education Anhui University Hefei China; ^2^ Nanotechnology Center Institute of Textiles & Clothing The Hong Kong Polytechnic University Hung Hom Kowloon Hong Kong; ^3^ Department of Materials Science and Engineering City University of Hong Kong Kowloon Hong Kong

**Keywords:** capacitor‐type anodes, hybrid micro‐supercapacitors, interlayer engineering, MXene, Zn^2+^ transfer kinetics

## Abstract

Micro‐supercapacitors are notorious for their low energy densities compared to micro‐batteries. While MXenes have been identified as promising capacitor‐type electrode materials for alternative zinc‐ion hybrid micro‐supercapacitors (ZHMSCs) with higher energy density, their tightly spaced layered structure renders multivalent zinc‐ions with large radii intercalation inefficient. Herein, through insertion of 1D core‐shell conductive BC@PPy nanofibers between MXene nanosheets, an interlayer structure engineering technique for MXene/BC@PPy capacitor‐type electrodes towards ZHMSCs is presented. Owing to simultaneously achieving two objectives: (i) widening the interlayer space and (ii) providing conductive connections between the loose MXene layers, enabled by the conductive BC@PPy nanospacer, the approach effectively enhances both ion and electron transport within the layered MXene structure, significantly increasing the areal capacitance of the MXene/BC@PPy film electrode to 388 mF cm^−2^, which is a 10‐fold improvement from the pure MXene film electrode. Pairing with CNTs/MnO2 battery‐type electrodes, the obtained ZHMSCs exhibit an areal energy density up to 145.4 μWh cm^−2^ with an outstanding 95.8% capacity retention after 25000 cycles, which is the highest among recently reported MXene‐based MSCs and approaches the level of micro‐batteries. The interlayer structure engineering demonstrated in the MXene‐based capacitor‐type electrode provides a rational means to achieve battery‐levelenergy density in the ZHMSCs.

## Graphical abstract

The demonstrated interlayer structure engineering synchronously realized the facilitated zinc‐ion and electron transfer kinetics between loose MXene nanosheets, resulting in enhanced charge storage capacity of MXene‐based capacitor‐type electrode toward hybrid micro‐supercapacitor with battery‐level energy density.

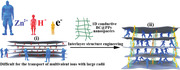

## 1 Introduction

Micro‐supercapacitors (MSCs) and micro‐batteries (MBs) based on non‐flammable aqueous electrolytes are considered to be promising next‐generation micro‐power solutions with high‐safety to support the rapid growing disciplines of wearable and implantable microelectronics.^[^ ^1^, ^2^, ^3^, ^4^, ^5^, ^6^, ^7^, ^8^, ^9^, ^10^, ^11^, ^12^ ^]^ Due to fast surface reaction on the electrodes, MSCs exhibit superior electrochemical performance compared to rechargable MBs in terms of a power density, rate capability, and cycle life.^[^ ^13^, ^14^, ^15^ ^]^ However, MSCs are notorious for their low energy density (≤10 μWh cm^−2^), which greatly restricts their wide spread adoption. In contrast, the charge storage mechanism of MBs is based on the insertion and extraction of ions inside the electrode material. Although this bulk reaction can achieve a high areal energy density, a low areal power density often results, mainly stemming from the sluggish insertion/extraction kinetics of ions with large ionic radii.^[^ ^16^, ^17^ ^]^ Therefore, as a well‐known challenge, neither conventional MSCs nor MBs can satisfy the demand for simultaneous high energy and power density. In order to combine the respective advantages of both types of energy storage devices, the hybrid micro‐supercapacitors (HMSCs), that pairing an ion intercalation battery‐type electrode and an ion adsorption capacitor‐type electrode have been recently reported.^[^ ^18^, ^19^, ^20^ ^]^ For this hybrid device, the amount of ions that the capacitor‐type electrode can effectively absorb/desorb fundamentally determines the energy density.

Amongst various ions serving as charge carriers, the use of monavalent Li^+^, Na^+^, and K^+^ included in inorganic electrolytes for most hybrid capacitors presents a great risk to safety and the environment.^[^ ^21^, ^22^, ^23^, ^24^ ^]^ In contrast, the systems based on multivalent ions in aqueous electrolytes (e.g., Zn^2+^) exhibit obvious advantages, such as improved safety, decreased cost, environmental friendliness, and easy of fabrication, which have attracted recent attentions.^[^ ^25^, ^26^, ^27^ ^]^ Currently, most of the employed ions absorption/desorption capacitor‐type electrodes in Zn^2+^ hybrid micro‐supercapacitors (ZHMSCs) are mainly carbonaceous materials such as commercial activated carbon,^[^ ^18^ ^]^ graphene,^[^ ^19^ ^]^ and carbon nanotubes.^[^ ^20^ ^]^ Although impressive progress has been achieved, the low specific capacitance of the carbon‐based materials based on the electric double layer storage mechanism and a small electrode thickness (to reduce bending strain for flexibility) have severely limited the energy density achieved in previously reported ZHMSCs. Therefore, overcoming the well‐known challenge of capacity mismatch between the capacitor‐type electrode and battery‐type electrode of ZHMSCs is the key to effectively removing the energy density bottleneck of ZHMSCs toward their commercial application.

**Scheme 1:**
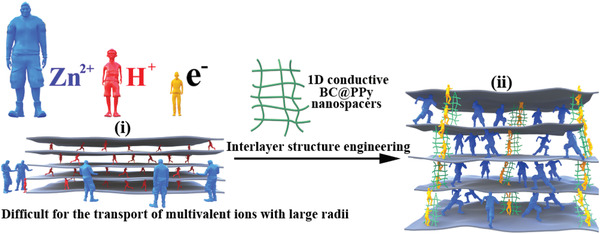
Illustration of the proposed interlayer structure engineering of MXene hybrid film: i) a notorious difficulty of intercalation of multivalent ions with large ionic radius, such as Zn^2+^. This problem has not been as severe for the more conventional and smaller hydrogen ion. ii) Insertion of 1D nanospacers to widen the space between MXene nanosheets, enabling the rapid and reversible insertion/deintercalation of large ions. The nanospacers also enhance electron transport between the loose MXene nanosheets.

Based on a newly discovered intercalation pseudocapacitance mechanism, in which the charge transport is not limited by solid phase diffusion, 2D transition metal carbide/nitride (MXenes) with a multi‐layered structure and achievable metallic conductivity can realize the storage of a large amount of charges in a short time via the effective ions intercalation between layers, which have been identified as an ideal candidate for new capacitor‐type electrode materials in ZHMSCs.^[^ ^28^, ^29^, ^30^ ^]^ Paradoxically, similar to other 2D materials, the strong van der Waals (vdW) forces and hydrogen bond interaction between the few‐layered MXenes greatly reduce the interlaminar space between the densely self‐assembled MXene nanosheets, severely blocking the rapid and reversible insertion/deintercalation of multivalent ions, which tend to have larger ionic radii and stronger coulomb interactions with the abundant negative terminal groups on the MXene surface (**Scheme** **1**i).^[^ ^31^, ^32^ ^]^ This problem has not been as serious for conventional MXene‐based MSCs based on the monovalent hydrogen ions, but greatly restricts breakthroughs in energy capacity of the promising MXene‐based ZHMSCs based on divalent zinc ions.

The method of nanospacer insertion between densely stacked few‐layered MXene nanosheets can effectively expand the interlaminar space, and thus improving the effectiveness of guest ions intercalation and charge storage of the MXene host electrodes.^[^ ^33^, ^34^, ^35^ ^]^ For example, nanospacers such as 1D bacterial cellulose,^[^ ^33^ ^]^ 1D carbon nanotubes,^[^ ^34^ ^]^ and 2D graphene^[^ ^35^ ^]^ have been used as nano intercalators of MXene film electrodes. However, the expansion of the interlayer space using the insulative and inactive bacterial cellulose deteriorates the electron transfer dynamics between the loose MXene nanosheets, resulting in limited improvement. Adding 1D conductive carbon nanotubes or 2D graphene with low specific capacity reduces the specific volumetric capacity of the host electrode. Thus, in order to realize multivalent ions based aqueous ZHMSCs with theoretically higher areal energy density, it is critical to well engineer the interlayer structure within the MXene capacitor‐type electrodes, which not only can facilitate more efficient Zn^2+^ transport (i.e., in greater amount and at higher rate), but also simultaneously allow unimpeded electron transport between the MXene layers, synergistically achieving a high charge storage capacity (Scheme 1ii).

To meet the demand for comfort in wearable applications, another well‐known challenge lies in achieving desirable mechanical properties such as stretchability and durability in the ZHMSCs.^[^ ^36^, ^37^, ^38^ ^]^ Currently, there are two dominant strategies to realize the stretchability of the available energy storage devices: i) directly depositing active film electrodes on natively stretchable substrate,^[^ ^39^ ^]^ and ii) using a strain‐tolerant design where rigid energy storage units and elastic interconnects are combined to realize stretchability on the system level.^[^ ^40^ ^]^ Although impressive progress has been made, both strategies have their respective limitations. Natively stretchable materials usually suffer from a decline of electrical conductivity under large deformation, resulting in a degradation of electrochemical performance under stretch. Strain‐tolerant designs, such as the serpentine pattern, also generally achieve high stretchability at the expense of large device size. Therefore, improvement in device architecture that can be stretched extensively but without degradation in electrochemical performance and sacrifice in device compactness is highly desirable.

Herein, for the first time, we present an interlayer structure engineering technique suitable for the MXene‐based capacitor‐type electrode of a hybrid micro‐supercapacitor. This approach enhances both ionic and electronic transport within the layered MXene structure, which provides an effective means to significantly improve energy density to the level exhibited in MBs. The technique is demonstrated through the design and fabrication of a ZHMSC based on a MXene/bacterial cellulose (BC)@polypyrrole (PPy) (MXene/BC@PPy) hybrid film capacitor‐type electrode. Due to the effective widening of the interlayer space through the insertion of elaborately designed 1D core–shell conductive BC@PPy nanosfibers between MXene nanosheets, ion transport between MXene nanosheets is significantly improved. Specifically, accelerated Zn^2+^ transfer kinetics of the obtained MXene/BC@PPy hybrid film electrode (1.67×10^−8^ cm^2^ s^−1^) compared to that of the pure MXene film electrode (0.0077×10^−8^ cm^2^ s^−1^) has been achieved. As an additional advantage, the inserted 1D BC@PPy nanofibers serve as conductive channels that enhance electron transport between the loose MXene nanosheets, as well as act as additional electroactive materials to further increase charge storage capacity. Thus, the fabricated MXene/BC@PPy hybrid film electrode exhibits a substantially increased areal capacitance of 388 mF cm^−2^ at the current density of 1 mA cm^−2^, which is more than 10 times the value of the pure MXene film electrode (38 mF cm^−2^) at the same current density. By further pairing with a carbon nanotubes(CNTs)@MnO_2_ battery‐type electrode, the fabricated ZHMSC achieved a maximum energy density of 145.4 μWh cm^−2^ at the current density of 0.75 mA cm^−2^, which is the highest energy density amongst recently reported state‐of‐the‐art MXene‐based MSCs, ZHMSCs with carbonous capacitor‐type electrode, and even comparable to zinc‐ion micro‐batteries (ZMBs). Furthermore, the ZHMSC exhibits an outstanding lifespan, achieving more than 95.8% retention in delivered capacitance after 25 000 cycles, much greater than the 65.9% from pure MXene film. Encouraged by the high energy efficiency of the ZHMSCs, a stretchable ZHMSC array (ZHMSCA) has also been realized through the seamless integration of ZHMSC elements with the intrinsically stretchable liquid metal (LM) interconnects. This array architecture is advantageous in that the ZHMSC elements are only responsible for energy storage while the intrinsically stretchable LM interconnects bear most of the applied stress during bend. The ZHMSCA delivers a stable and tunable output voltage and energy in the range from 1.9 to 7.6 V and 122.5 to 128.4 μWh even under a large elongation up to 400%, demonstrating great potential as a compatible deformable micro‐power source for wearable and implantable microelectronics.

## 2 Results and Discussion

### 2.1 Interlayer Structure Engineering of MXene‐Based Capacitor‐Type Electrode

**Figure 1:**
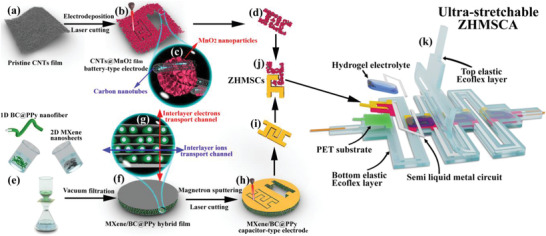
Illustration of the fabrication process of the ultra‐stretchable ZHMSCA: a) Pristine CNTs film consisting of randomly oriented carbon nanotubes; b) Electrodeposition of MnO_2_ nanoparticles on the surface of CNTs film toward CNTs@MnO_2_ hybrid film that was further laser‐cutted into d) interdigital CNTs@MnO_2_ battery‐type electrode; e) Mixed vacuum‐filtration of 2D MXene nanosheets and 1D BC@PPy nanofibers into f) MXene/BC@PPy hybrid film that was further h) laser‐cutted into i) interdigital capacitor‐type electrode; j) Assembled ZHMSC; k) Decomposition diagram of the ultra‐stretchable ZHMSCA; c,g) Enlarged schematic diagrams of the microstructure.

The fabrication procedure of an ultra‐stretchable ZHMSCA based on the island‐bridge architecture was schematically illuminated in **Figure** **1**, with the following steps: i) fabricating the interdigital CNTs@MnO_2_ battery‐type electrode (Figure 1d) and MXene/BC@PPy capacitor‐type electrode (Figure 1i) via laser‐cutting the CNTs@MnO_2_ hybrid film (Figure 1b) and MXene/BC@PPy hybrid film (Figure 1f), ii) pairing the two aforementioned electrodes into ZHMSC active units (Figure 1j), iii) fixing the active units on a stiffer PET film supporter via double‐sided adhesive tape and interconnecting the “islands” regions with screen‐printed LM “bridges” on a bottom elastic supporter layer (Figure 1k), and viii) coating the hydrogel electrolyte and uncured silicone as the top encapsulation layer, and v) curing of the top silicone encapsulation layer, thereby completing the ZHMSCA.

**Figure 2:**
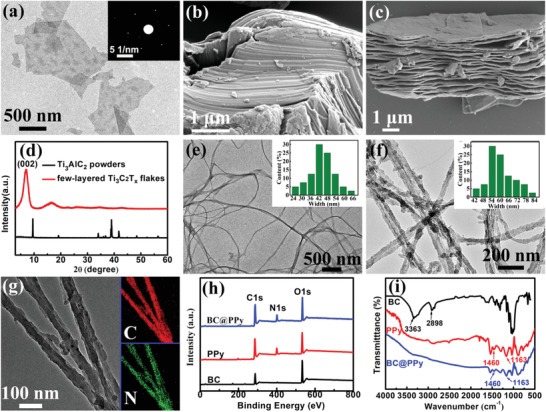
a) A TEM image of the obtained few‐layered Ti_3_C_2_Tx nanosheets (inset is the corresponding SAED pattern); b) A SEM image of the Ti_3_AlC_2_ particles and c) obtained multi‐layered Ti_3_C_2_Tx; d) XRD results of the Ti_3_AlC_2_ and few‐layered Ti_3_C_2_Tx nanoflakes; Typical TEM images of e) pristine BC nanofibers and f) BC@PPy nanofibers (the insets show the corresponding dimension distribution); g) Elemental mappings of the TEM picture of C, and N on the 1D BC@PPy nanofibers; Comparative analysis of h) XPS survey spectrum and i) FTIR spectra of the samples.

The key MXene/BC@PPy hybrid film (Figure 1f) was prepared via vacuum‐filtration of a mixed solution containing 1D BC@PPy conductive nanofibers and 2D few‐layered Ti_3_C_2_Tx nanosheets (Figure 1e). The Ti_3_C_2_Tx nanosheets (Figure 2a) were prepared by etching the Ti_3_AlC_2_ MAX precursor (**Figure** **2**b), followed by ultrasonication exfoliation of the obtained multi‐layered Ti_3_C_2_Tx (Figure 2c). The vanishing of the typical Ti_3_AlC_2_ MAX phase diffraction peaks and generation of a characteristic peak located at ≈6.4° (Figure 2d) indicate the successful preparation of few‐layered Ti_3_C_2_Tx nanosheets.^[^ ^6^, ^34^ ^]^ The 1D BC@PPy core–shell conductive nanofibers were prepared via the simple chemical oxidative polymerization of pyrrole monomers on the surface of the 1D BC nanofibers (Figure 2e).^[^ ^41^ ^]^ The diameter of the pristine BC nanofibers is in the range of 24 to 66 nm (inset in Figure 2e). After coating treatment, the diameter of the as‐obtained 1D BC@PPy nanofibers is significantly increased into the range of 42 to 84 nm (Figure 2f). The uniform distribution of nitrogen signal observed on the surface of the 1D BC@PPy nanofibers in the elemental mapping images (Figure 2g) intuitively shows the existence of a uniform PPy shell. Both the X‐ray photoelectron spectroscopy (XPS) and infrared spectrum (IR) further reveal the characteristic peaks belonging to nitrogen (402.0 eV) and the C─N bond (the peaks at 1460 and 1163 cm^−1^ belonging to C‐N stretching vibration in the pyrrole ring and C‐N in‐plane ring deformation, respectively).^[^ ^42^ ^]^ In comparison with the spectra of pristine BC nanofibers and pure PPy samples, the successful deposition of conformal PPy conductive shell on the surface of the BC nanofibers is fully evident.

**Figure 3:**
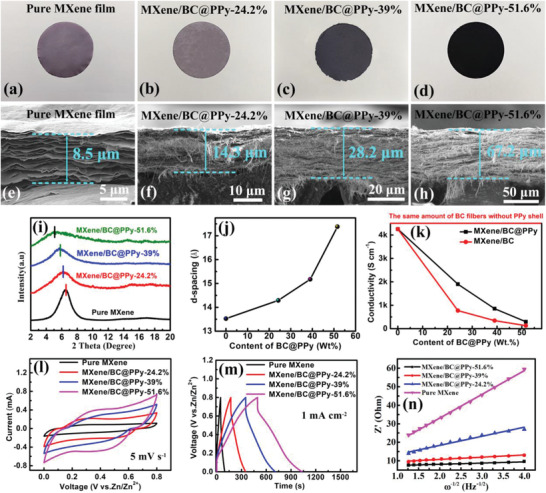
a–d) Optical photos, e–h) corresponding SEM images of the evolution in cross‐section morphology, and i) evolution of XRD patterns of the as‐fabricated MXene/BC@PPy hybrid films with different mass percentage of BC@PPy nanofibers; j) Evolution of the d‐spacing and k) electrical conductivity in the MXene/BC@PPy hybrid films versus the content of BC@PPy and pristine BC without PPy shell; Corresponding comparison of l) CV curves under the same scanning speed, m) GCD curves at the same current density; n) Plots of Z’ against *ω* ^−1/2^.

The evolution of morphology and enlargement of interlayer spacing of the fabricated MXene/BC@PPy hybrid films (**Figure** **3**a–d) with respect to increasing mass percentage of the homogeneously inserted BC@PPy nanofibers were characterized by SEM (Figure 3e–h) and X‐ray diffraction spectroscopy (XRD, Figure 3i), respectively. All the fabricated MXene/BC@PPy hybrid films such as MXene/BC@PPy‐51.6% were labelled according to the ratio between MXene and BC@PPy in the synthesis as shown in the Experimental Section in Supporting Information. The cross‐sectional SEM images clearly show that the obtained free‐standing and binder/conductive‐additive free MXene/BC@PPy hybrid films are mainly composed of the well‐aligned stacked few‐layered Ti_3_C_2_Tx nanosheets, between which the 1D BC@PPy nanofibers are evenly inserted. In addition, the characteristic (002) peak sharply shifted from ≈6.4° in the pure MXene film to ≈5.15° in the MXene/BC@PPy‐51.6% hybrid film with respect to increasing mass percentage of the added BC@PPy nanospacer. This corresponds to an expansion in d‐spacing from 13.82 to 17.16 Å (Figure 3j). More importantly, the evenly inserted 1D BC@PPy nanofibers not only can serve as interlayer scaffold to expand the interlayer space, building fast ions diffusion channels, but also act as interlayer conductive channels to facilitate the electron transport between the poorly contacted MXene nanosheets. As shown in Figure 3k, with an increase in the mass percentage of BC@PPy nanofibers, the conductivity of the MXene/BC@PPy hybrid film gradually decreases. However, at any given mass percentage, the conductivity is still higher than that of the film without the PPy shell.

The Zn^2+^ storage capacity of the MXene/BC@PPy capacitor‐type electrode was first evaluated by pairing with a Zn foil electrode in a planar two electrode configuration (Figure S1a, Supporting Information). The detailed size of the cell is illuminated in the Figure S1b (Supporting Information). Figure 3l,m are the corresponding CV and GCD curves of the MXene/BC@PPy capacitor‐type electrode with different mass percentages of the BC@PPy nanospacer, respectively. Evidently, both the response current at the same scan rate and running time at the same current density gradually improve with respect to increasing mass percentage of BC@PPy nanospacer within MXene/BC@PPy hybrid electrodes. Remarkably, a more than tenfold increase in areal capacity (388 mF cm^−2^) compared to that of pure MXene film electrode (38 mF cm^−2^) has been achieved using the MXene/BC@PPy‐51.6% hybrid film at a current density of 1 mA cm^−2^. In order to determine whether the expanded interlayer spacing indeed has an impact on the transfer kinetics of Zn^2+^ within the MXene/BC@PPy host electrodes, which affects the charge storage capacity, EIS measurement was further performed. Figure S2 (Supporting Information) and Figure 3n show the Nyquist plots and plots of *Z*’ versus *ω* ^−1/2^ for the MXene/BC@PPy hybrid film electrodes with different mass percentages of the BC@PPy nanospacer, respectively. The derived kinetic parameters are shown in **Table** **1**. Evidently, a sharp increase in the Zn^2+^ diffusion coefficient within the MXene/BC@PPy hybrid film electrodes has been observed with the increasing interlayer space derived from the homogeneous insertion of BC@PPy nanospacers. A maximal Zn^2+^ diffusion coefficient of 1.67 × 10^−8^ cm^2^ s^−1^ has been achieved for the MXene/BC@PPy‐51.6% hybrid film electrode, which is nearly 217 times that of the pure MXene film electrodes (0.0077 × 10^−8^ cm^2^ s^−1^). Furthermore, generally, the larger slope (*σ*, Warburg factor) of *Z*’ verse *ω* ^−1/2^ implies a smaller diffusion coefficient (inversely proportional to *σ* ^−2^), thus slower ions transfer kinetics.^[^ ^43^ ^]^ From Table 1, it is evident that the MXene/BC@PPy‐51.6% hybrid film electrode demonstrates a significant reduction in *σ* to 0.91, compared to 13.36 of pure MXene film electrode, also suggesting improved Zn^2+^ diffusion. Coupling the electrochemical investigations with structural analysis, results strongly suggest that the expansion of the interlayer space between few‐layered MXene nanosheets via intercalation of BC@PPy nanospacers is an effective optimization strategy to achieve faster Zn^2+^ diffusion, resulting in enhanced areal charge capacity of the MXene/BC@PPy hybrid film electrode.

**Table 1:** Kinetic parameters of MXene/BC@PPy hybrid film electrodes with different mass percentages of BC@PPy nanofibers

Key parameter	Pure MXene	MXene/BC@PPy‐24.2%	MXene/BC@PPy‐39.0%	MXene/BC@PPy‐51.6%
d‐spacing [Å]	13.82	14.31	15.43	17.16
*σ* [Ω Hz^1/2^]	13.36	3.38	1.03	0.91
Zn^2+^ diffusion coefficient [10^−8^ cm^2^ s^−1^ ]	0.0077	0.12	1.30	1.67

**Figure 4:**
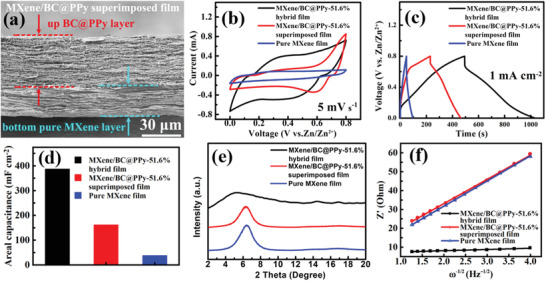
a) Cross‐section SEM image of MXene/BC@PPy superimposed film electrode; Comparison of the b) CV curves at the same scanning speed, c) GCD profiles at the same current density, and d) the areal capacitance of ZHMSCs employing MXene/BC@PPy hybrid film, MXene/BC@PPy superimposed film, and pure MXene film; e) Comparison of the XRD patterns of the MXene/BC@PPy hybrid film, MXene/BC@PPy superimposed film, and pure MXene film; f) Plots of *Z*’ against *ω* ^−1/2^ for the samples.

As an additional advantage, PPy is also electroactive for the Zn^2+^ storage in the ZHMSCs.^[^ ^44^ ^]^ Thus, to probe further into the contribution stemming from improved Zn^2+^ transfer kinetics toward enhancing charge storage capacity of the MXene/BC@PPy hybrid film electrode, MXene/BC@PPy composite film with a simple superimposed structure (**Figure** **4**a) obtained by sequential filtration of a MXene and BC@PPy dispersion was prepared as a reference. Evidently, a larger response current at the same scan rate (Figure 4b) and a longer running time at the same current density (Figure 4c) have been observed for the MXene/BC@PPy‐51.6% superimposed film electrode than those of pure MXene film electrode, reflecting an increased charge storage capacity derived from the electroactive PPy shell. Remarkably, both of the parameters are less than those of the MXene/BC@PPy‐51.6% hybrid film electrode. According to computational results, the MXene/BC@PPy superimposed film electrode acquires a maximum areal capacitance of 162.3 mF cm^−2^ at 1 mA cm^−2^, which only reaches 42% of the value from the MXene/BC@PPy hybrid film at the same current density (Figure 4d). In addition, the d‐spacing (13.93 Å) from XRD pattern (Figure 4e) and derived Zn^2+^ diffusion coefficient (0.0079 × 10^−8^ cm^2^ s^−1^) from the EIS measurement (Figure 4f) and for the MXene/BC@PPy superimposed film electrode are close to that of pure MXene film electrode (13.82 Å and 0.0077 × 10^−8^ cm^2^ s^−1^), but significantly lower than that of the MXene/BC@PPy hybrid film electrode (17.16 Å and 1.67 × 10^−8^ cm^2^ s^−1^). This suggests that the obvious difference in areal capacitance between the two electrodes can be attributed to the crucial role played by the proposed interlayer structure engineering, resulting in accelerated Zn^2+^ diffusion and alleviated deterioration of electron transport between MXene nanosheets. Combined with the other advantages of the PPy active shell such as inherent storage capacity, a substantial increase in areal capacity has been achieved for the MXene/BC@PPy hybrid film electrode, which can be directly employed for robust and high‐areal‐capacity capacitor‐type anode in the following demonstrated ZHMSCs.

### 2.2 CNTs@MnO_2_ Battery‐Type Electrode

**Figure 5:**
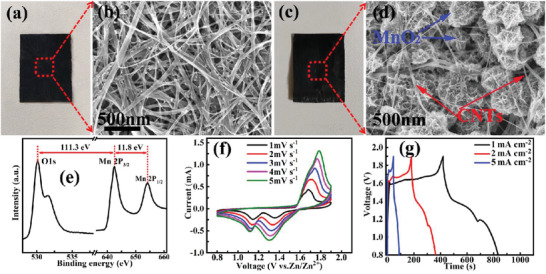
Photographs and SEM images of a,c) the pristine CNTs film and b,d) the CNTs/MnO_2_ film; e) XPS spectrum of the electrodeposited MnO_2_ on the CNTs film; f) the CV and g) the GCD curves of the CNTs/MnO_2_ film electrode.

Characterized by its high theoretical capacity (≈300 mAh g^−1^), abundance as a resource, and environmental benignity, MnO_2_ is a promising candidate for battery‐type electrode material in ZHMSCs.^[^ ^45^, ^46^ ^]^ However, the intrinsically poor electron/ion transport of pristine manganese oxide (10^−5^–10^−6^ S cm^−1^) and the high impedance associated with intercalation redox reactions of oxides are well‐known limitations,^[^ ^5^ ^]^ greatly restraining utilization of MnO_2_‐based battery‐type electrode in ZHMSCs for higher energy efficiency. In the fabricated ZHMSC, in order to address the innate disadvantage of MnO_2,_ freestanding and highly conductive CNTs film (**Figure** **5**a) consisting of a 3D porous conductive framework of interlaced carbon nanotubes (Figure 5b) was employed to electrodeposit MnO_2_ nanoparticles for the battery‐type film electrode (Figure 5c). The preparation method provides an intimate direct contact of MnO_2_ with the highly conductive CNTs, without the use of any binder or conductive additives (Figure 5d). XPS characterization was employed to reveal the chemical composition of the electrodeposited manganese oxide (Figure 5e). Two peaks at 642.4 and 654.2 eV, respectively belonging to the binding energies of Mn 2p 3/2 and 2p 1/2, are clearly observed.^[^ ^47^ ^]^ The spin‐energy gap (11.8 eV) between the peaks indicates that the main valence state of Mn is +4.^[^ ^48^ ^]^ Moreover, the energy gap (111.3 eV) between O 1s (Mn‐O‐Mn) and Mn 2p 3/2 also indicates that Mn mainly exists in the form of 4‐valent ions in the sample.^[^ ^49^ ^]^ Shown by the above analysis, the electrodeposited nanoparticles are mainly MnO_2_ and has a flower‐like morphology. The CNT fibers around the electrodeposited MnO_2_ nanoparticles (Figure 5d) can serve as an efficient electron transfer path to effectively improve the electron transport kinetics during the charge storage process (Figure 1c), resulting in stable and reversible CV curves with obvious redox peak pairs of a narrow half‐width (Figure 5f), which highlights the excellent reaction kinetics and redox efficiency of the CNTs@MnO_2_ battery‐type film electrode. In addition, the stable and reversible CV curves in the potential range of 0.8–1.9 V also prove a stable voltage window of 1.1 V. In contrast, the MXene/BC@PPy hybrid film electrodes can function well in the potential range of 0–0.8 (Figure 3l), representing a stable voltage window of 0.8 V. Furthermore, calculated from the GCD curves (Figure 5g), the as‐obtained CNTs@MnO_2_ hybrid film electrode can deliver a high areal capacitance of 186 mF cm^−2^ at the current density of 2 mA cm^−2^ (electrodeposition time: 200 s and mass loading of MnO_2_: 0.33 mg cm^−2^). Thus, combined with a well matched voltage window range, a suitable balance in the charge storage capacity can be achieved between the as‐obtained CNTs@MnO_2_ battery‐type film electrode and the MXene/BC@PPy capacitor‐type film electrode (275 mF cm^−2^ at the same current density of 2 mA cm^−2^, Figure S3: Supporting Information) on the basis of well‐known formulae of Qp=Qn, Qp=CpVp, Qn=CnVn (Qp/Qn, Cp/Cn, and Vp/Vn representing the charge storage capacity, delivered capacitance, and voltage window of the positive/negative electrodes, respectively), which results in high energy density when used in ZHMSCs.

### 2.3 Zinc‐Ion Hybrid Micro‐Supercapacitors

**Figure 6:**
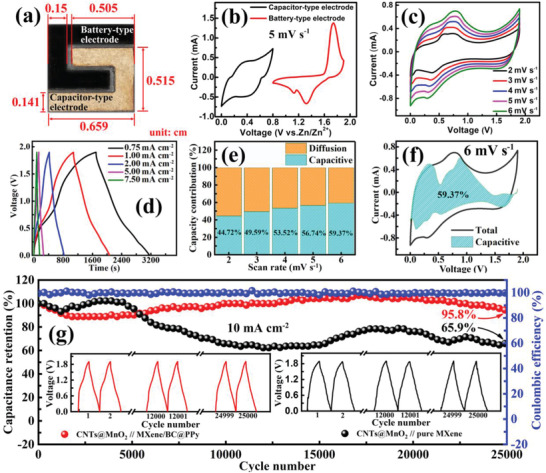
a) Typical photograph of a ZHMSC unit assembled based on MXene/BC@PPy capacitor‐type electrode and CNTs@MnO_2_ battery‐type electrode; b) CV curves of the MXene/BC@PPy capacitor‐type electrode and CNTs@MnO_2_ battery‐type electrode; c) CV curves of the ZHMSC at different scanning speed; d) GCD curves at different current densities; e) Pseudocapacitive contributions of MXene/BC@PPy capacitor‐type electrode at different scanning speed; f) CV curve at 6 mV s^−1^ with a marked region indicating the capacitive controlled contribution of about 59.37%; g) Cycling stability test of the ZHMSC based on MXene/BC@PPy and pure MXene capacitor‐type electrodes with the same CNTs@MnO_2_ battery‐type electrode.

Based on the well‐matched capacitor‐type anode and battery‐type cathode (**Figure** **6**b), a ZHMSC unit was assembled (Figure 6a) with a 2 m Zn(CF_3_SO_3_)_2_‐0.1 m MnSO_4_/polyacrylamide (PAM) hydrogel as the solid‐state electrolyte. As shown in Figure 6c, the hybrid device exhibits stable and reversible CV curves with obvious redox peaks at the scanning rates of 2–6 mV s^−1^ in a wide potential range of 0–1.9 V. Calculation based on the GCD curves (Figure 6d) indicates that the ZHMSC can deliver a maximum areal capacitance of 290 mF cm^−2^ at a current density of 0.75 mA cm^−2^. Together with the wide voltage window, a high areal energy density up to 145.4 μWh cm^−2^ at the current density of 0.75^ ^mA cm^−2^, corresponding to a minimum areal power density of 0.36 mW cm^−2^ (28.1 mWh cm^−3^ for volumetric energy density and 69.5 mW cm^−3^ for volumetric power density corresponding to the total volume of positive and negative electrodes), can be achieved in the ZHMSC. When the current density is increased 10 times, 54.8% of the energy density (79.72 μWh cm^−2^ at 7.5 mA cm^−2^), corresponding to a maximal areal power density of 3.78 mW cm^−2^ (15.4 mWh cm^−3^ for volumetric energy density and 729.7 mW cm^−3^ for volumetric power density corresponding to the total volume of positive and negative electrodes), can be achieved, showing good rate capability. As is well‐known, the response current *i* that corresponds to each voltage value observed in the CV curve consists of two components, due to: i) the pseudocapacitive effect, and ii) the diffusion‐controlled insertion reaction, which can be described as *i* = *k*_1_ *v* + *k*_2_ *v* ^1/2^.^[^ ^12^ ^]^ The terms *k*_1_ *v* and *k*_2_ *v* ^1/2^describe the pseudocapacitive effect and diffusion‐limited process, respectively.^[^ ^50^ ^]^ Thus, quantifying the respective contribution ratio from the two components can provide insights into the energy storage mechanism of the hybrid device. Evidently, with the increase of the scanning speed from 2 to 6 mV s^−1^, the contribution ratio from capacitive effect gradually increases from 44.7% to 59.4% (Figure 6e, shaded area). This indicates that along with the increase of the scanning speed, the contribution derived from capacitive effect in the total capacity gradually dominants.^[^ ^51^ ^]^ Furthermore, the enlarged interlayer structure of the MXene/BC@PPy‐51.6% electrode enables the ZHMSC to achieve a better cycle stability of 95.8% versus 65.9% from pure MXene electrode based device in areal capacitance retention after 25 000 cycles (Figure 6g). **Table** **2** and Figure S4 (Supporting Information) (Ragone plot) show a performance comparison amongst state‐of‐the‐art MXene‐based MSCs, ZHMSCs based on carbon‐based capacitor‐type electrode, and ZMBs. The fabricated ZHMSC shows an unprecedented level of performance, with the most competitive power density and energy density amongst recently reported MXene‐based MSCs,^[^ ^33^, ^42^, ^52^, ^53^, ^54^, ^55^, ^56^, ^57^ ^]^ as well as an energy density that remarkably approaches that of a VO_2_‐MWCNTs//Zn based ZMB.^[^ ^12^, ^58^ ^]^

**Table 2:** Performance comparison of recently reported state‐of‐the‐art MXene‐based MSCs, ZHMSCs based on carbon‐based capacitor‐type electrode, and ZMBs. (*) Symmetrical MSCs; (⁑) Asymmetric MSCs; (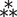) ZHMSCs; (†) ZMBs

Electrodes	Classification	Voltage Windows [V]	Areal capacitance [mF cm^−2^]	Areal energy density [uWh cm^−2^]	Areal power density [mW cm^−2^]	Ref.
CNTs@MnO_2_//MXene/BC@PPy	( 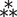 )	0–1.9	290	Maximum:145.4 Minimum: 79.72	Maximum:3.78 Minimum: 0.36	This work
Be^2+^‐MXene// Be^2+^‐MXene	(*)	0–0.6	77.2	3.86	0.12	^[^ ^52^ ^]^
MXene/BC//MXene/BC	(*)	0–0.6	112.2	5.54	0.114	^[^ ^33^ ^]^
Screen‐printed MXene//MXene	(*)	0–0.6	158	1.64	0.78	^[^ ^56^ ^]^
CNTs@PPy//Ti_3_C_2_Tx	(⁑)	0–1.4	150.22	40.49	0.26	^[^ ^42^ ^]^
RuO_2_//Ti_3_C_2_Tx	(⁑)	0–1.5	60	19	1.5	^[^ ^53^ ^]^
Co‐Al‐LDH//Ti_3_C_2_Tx	(⁑)	0.4–1.45	40	8.84	0.23	^[^ ^54^ ^]^
Polymer‐MXene//MnO_2_	(⁑)	0–1.6	69.5	Maximum: 27.29 Minimum: 15.1	Maximum: 3.59 Minimum: 0.204	^[^ ^55^ ^]^
Ti_3_C_2_Tx//MnO_2_	(⁑)	0–2.0	295	Maximum: 162 Minimum: 133	Maximum: 54 Minimum: 2.7	^[^ ^57^ ^]^
AC//Zn	( 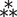 )	0.5–1.5	1297	Maximum: 115.4 Minimum: 89.0	Maximum: 3.9 Minimum: 0.16	^[^ ^18^ ^]^
CNT//Zn	( 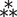 )	0.2–1.8	83.2	Maximum: 29.6 Minimum: 23.1	Maximum: 8 Minimum: 0.8	^[^ ^20^ ^]^
VO_2_‐MWCNTs//Zn	(†)	0–2.0	N.A.	Maximum: 188.8 Minimum: 70.3	Maximum: 0.61 Minimum: 0.09	^[^ ^12^ ^]^
CNTs/TPU/PANI//CNTs/TPU/Zn	(†)	0–2.0	N.A.	250	0.2	^[^ ^58^ ^]^

### 2.4 Ultra‐Stretchable ZHMSC Array Based on Liquid Metal Island‐Bridge Device Architecture

**Figure 7:**
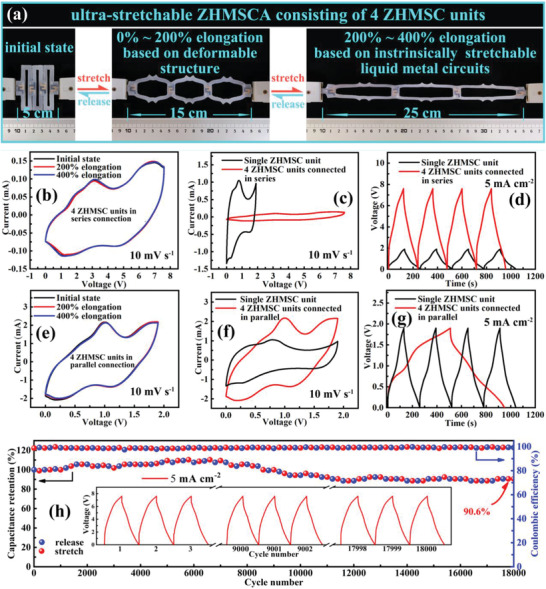
a) Optical photos of the ZHMSCA consisting of 4 ZHMSC units, connected in series or parallel, stretched from 0% to 200% and 400% elongation; b,e) Real‐time recorded CV curves; c,f) CV curves of ZHMSCA and single ZHMSC unit at the same scanning speed of 10 mVs^−1^, and d,g) GCD curves of the ZHMSCA and single ZHMSC unit at the same current density of 5.0 mA cm^−2^; h) Cycling stability test, subjecting the ZHMSCA device to stretching state with 400% uniaxial strain for every 200 charge‐discharge cycles.

Demand for comfort and esthetics in flexible/wearable electronics necessitates excellent mechanical properties in micro energy storage devices.^[^ ^59^, ^60^ ^]^ To this end, we present the “island‐bridge” device architecture, consisting ZHMSC “islands” with the intrinsically stretchable LM “bridges”, realizing the highly stretchable ZHMSC array (ZHMSCA). As shown in **Figure** **7**a, the ZHMSCA consisted of four fixed ZHMSC islands, interconnected by screen‐printed stretchable LM bridges with mechanically deformable structure, all of which are packaged in an elastic silicon matrix. Within the strain range of 0–200%, the LM bridge interconnects are geometrically deformed, absorbing most of the stress. As shown in the in situ recorded CV curves, electrochemical performance is insensitive to the applied stress during stretch (Figure 7b,e). When the strain exceeds 200%, the intrinsically stretchable LM interconnects begin to stretch themselves, which also results in negligible changes in the in‐situ recorded CV curves even for strain up to 400%. In both stain regimes, the interconnect absorbs the majority of the stress thus protecting the ZHMSC elements. Furthermore, the ZHMSCs can be connected in series or in parallel through the island‐bridge architecture, achieving a tailorable overall output voltage and capacitance. As shown in Figure 7c,d, four series‐connected ZHMSC elements within the ZHMSCA can output a high voltage up to 7.6 V. Meanwhile, the ZHMSCA consisting of four parallel‐connected ZHMSCs can deliver a maximum capacitance of 244.4 mF, nearly four times that of a single ZHMSC element (70.4 mF) at the current density of 5 mA cm^−2^ as shown in Figure 7f,g. Thus, through different series‐parallel connections, ZHMSCAs based on the island‐bridge architecture have been tested to output a voltage range of 1.9 to 7.6 V and an energy range of 122.5 to 128.4 μWh.

The ZHMSCA also shows a high durability under repeated deformation testing. As shown in Figure 7h, after experiencing persistent stretching deformation under 400% elongation more than 18 000 cycles (stretch every 200 cycles), the capacitance retention of the ZHMSCA still exceeds 90.6%, indicating outstanding tensile durability. As an application test, a ZHMSCA consisting four ZHMSC elements in a series connection can reliably power a digital watch under repeated stretching deformation between 0% and 400% elongation, as shown in the Video S1 in the Supporting Information. The above extensive characterization fully demonstrates the excellent electrochemical stability, stretchability, as well as integration capacity of the devices, confirming their strong technical strength for next‐generation deformable micro‐power solution.

### 2.5 Quantitative Analysis of Tensile Properties of the ZHMSCA under Stress

**Figure 8:**
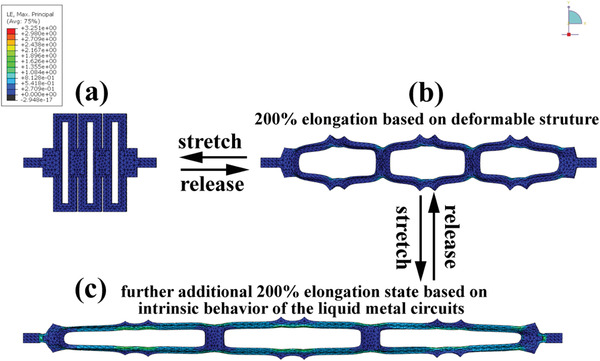
Finite element analysis (FEA) simulation results of strain distribution on the ZHMSCA under a) initial state (0% elongation), b) 200% elongation, and c) 400% elongation.

For quantitative insights into the effectiveness of the island‐bridge architecture under stress, finite element analysis (ABAQUS, Dassault Systèmes) simulation has been performed. As shown in **Figure** **8**b, under a 200% strain (simulated at uniaxial strain), the island‐bridge interconnected structure can effectively dissipate the strain induced on the device via the geometrical extension of the interconnected pattern. Evidently, the induced strain mainly concentrated on the deformable interconnection arms (ɛ_max_=187%), in contrast to ɛ_max_=19.9% on the rigid ZHMSC islands. Furthermore, when the ZHMSCA was under 400% strain, the intrinsically stretchable LM bridges begins to stretch themselves, absorbing any further increases in stress, thus protecting the rigid ZHMSC elements as shown in Figure 8c. This is due to the significantly lower Young's modulus of the Ecoflex substrate (69 kPa) compared to the stiffer polyethylene terephthalate film substrate that hardly deforms (3700 MPa, 0.1 mm in thickness).^[^ ^61^ ^]^ In fact, at 400% strain, the LM interconnects absorb 325.1% of the strain while the ZHMSC elements only bare 67.6%, which is merely 20% of the total load, effectively protecting the active elements. Evidently, experimental characterizations and theoretical calculations are highly correlated and reveal the mechanism by which electrochemical stability can be achieved even under extensive stretch.

## 3 Conclusion

This paper presents an interlayer structure engineering technique that simultaneously achieves optimization of interlayer structure and components of the MXene/BC@PPy hybrid capacitor‐type film electrode. Electrochemical tests show that the accelerated Zn^2+^ diffusion and alleviated deterioration of electrons transmission stemming from the interlaminar insertion of 1D conductive BC@PPy nanospacer between MXene nanosheets, as well as additionally loaded PPy active shell synergistically contribute to the acquired superior areal capacitance and durability of the MXene/BC@PPy hybrid capacitor‐type film electrode. By further being paired with a matching CNTs@MnO_2_ battery‐type electrode, ZHMSCs acquiring a maximum energy density of 145.4 μWh cm^−2^ at a high areal power density of 0.36 mW cm^−2^ and an outstanding lifespan (95.8% capacity retention after 25 000 cycles) were fabricated. Moreover, rooting in a novel “island‐bridge” architecture that combines the inherent merits of intrinsically stretchable liquid metal circuits with high electrical stress endurance and mechanically deformable structure with high strain accommodation, a stretchable ZHMSCA was developed, which can realize a tunable output voltage and energy in the range from 1.9 to 7.6 V and 122.5 to 128.4 μWh, while maintaining the electrochemical performance well under a high tensile deformation up to 400% elongation. The demonstrated interlayer structure engineering of the MXene hybrid film provides a promising strategy to synergistically address the challenge of sluggish transfer kinetics of divalent ions within the MXene host electrodes, while alleviating the reduction of electrical conductivity between loose MXene nanosheets, resulted in boosted charge storage capacity of MXene based capacitor‐type electrode for ZHMSCA with battery‐level energy density toward high‐performance stretchable compatible micro‐power sources in wearable microelectronics.

## Conflict of Interest

The authors declare no conflict of interest.

## Supporting information

Supporting Information

Supplemental Video 1

## Data Availability

Research data are not shared.
