# Supplementary material for: Interlayer Structure Engineering of MXene‐Based Capacitor‐Type Electrode for Hybrid Micro‐Supercapacitor toward Battery‐Level Energy Density
Source: Adv Sci (Weinh). 2021 Jun 17;8(16):2100775. doi: 10.1002/advs.202100775 (PMC8373094; doi:10.1002/advs.202100775)
Supplement: Supplementary file 1 — Supporting Information [file ADVS-8-2100775-s001.pdf]

© 2021 Wiley-VCH GmbH

ADVANCED SCIENCE
Open Access

Supporting Information

for *Adv. Sci.*, DOI: 10.1002/advs.202100775

Interlayer structure engineering of MXene-based capacitor-type electrode for hybrid micro-supercapacitor towards battery-level energy density

Wenxiang Cheng, Jimin Fu, Haibo Hu, and Derek Ho**

Supporting Information

Interlayer structure engineering of MXene-based capacitor-type electrode for hybrid micro-supercapacitor towards battery-level energy density

Wenxiang Cheng,[+] Jimin Fu,[+] Haibo Hu,* and Derek Ho*

Mr. W. X. Cheng[+] and Dr. H. B. Hu
School of Physics and Materials Science, Key Laboratory of Structure and Functional Regulation of Hybrid Materials, Anhui University, Hefei, China;
Email: haibohu@ahu.edu.cn

Dr. J. M. Fu[+]
Nanotechnology Center, Institute of Textiles & Clothing, The Hong Kong Polytechnic University, Hung Hom, Kowloon, Hong Kong, China;

Dr. H. B. Hu and Dr. D. Ho
Department of Materials Science and Engineering, City University of Hong Kong, Kowloon, Hong Kong, China;
Email: derekho@cityu.edu.hk

[+] These authors contributed equally to this work.

Keywords: interlayer engineering, MXene, capacitor-type anode, Zn^{2+} transfer kinetics, hybrid micro-supercapacitor

Experimental Section

Synthesis of multilayered Ti_3C_2Tx:

Multilayered Ti_3C_2Tx was synthesized through etching pristine Ti_3AlC_2 powder to remove the Al-layers. Typically, 1 g LiF was dissolved in 16 mL 9 M HCl. Then 0.45 g Ti_3AlC_2 powder was added into the above etching solution and kept at 40 °C for 48 h while stirring. Afterward, the solid residue was washed with deionized water and then collected until the pH of the supernatant was above 6. After drying, the multilayered Ti_3C_2Tx was obtained.

Preparation of few-layered Ti_3C_2Tx nanosheets:

The above-obtained multilayered Ti_3C_2Tx powder (1 g) was then dispersed in 150 mL deionized water and sonicated for 1 h under ice bath. After centrifugation at 4000 rpm for 5 min to remove the solid residue, the dark green supernatant containing the fully delaminated few-layered Ti_3C_2Tx nanosheets was collected for further use, whose concentration was determined by filtering a known volume of the suspension and measuring the weight of the film after vacuum drying.

Preparation of 1D conductive bacterial cellulose (BC)@polypyrrole (PPy) nanofibers with core-shell structure:

Typically, 2.1 mL pyrrole monomer was firstly dispersed in 6 mL absolute ethanol. Then the mixed solution was added into 50 ml BC solution (1.5 mg mL^{-1}) in an ice bath. Next, the prepared hydrochloric acid solution (50 ml, 2 M) containing 0.84 g ferric chloride hexahydrate was added into the above suspension slowly under vigorous stirring. After reaction at 0 °C for 9 h, the 1D conductive BC@PPy nanofibers with core-shell structure were obtained via vacuum filtration.

Fabrication of MXene/BC@PPy hybrid films with different weight content of 1D BC@PPy nanofibers:

Typically, the obtained BC@PPy nanofibers were re-dispersed into 200 ml DI water in the presence of sodium dodecyl benzene sulfonate (SDBS, 1 mg mL^{-1}) under ultrasonic treatment to obtain a stable suspension of BC@PPy nanofibers (0.6 mg mL^{-1}). Then the MXene suspension (60 ml, 0.5 mg ml^{-1}) was dropwise added into the BC@PPy nanofibers suspension with different volumes (16 ml for MXene/BC@PPy-24.2%, 32 ml for MXene/BC@PPy-39%, and 53 ml for MXene/BC@PPy-51.6%, 0.6 mg mL^{-1}). After a quick magnetic stirring for 1 h, the MXene/BC@PPy hybrid films with homogeneously intercalated 1D conductive BC@PPy nanofibers of different weight content from 0%-51.6% were obtained by vacuum-filtrating through a cellulose filter membrane for further use.

Fabrication of flexible and free-standing CNTs@MnO_2 hybrid film:

The flexible and free-standing CNTs@MnO_2 film electrode was prepared via the electrodeposition, which was accomplished in a three-electrode system by using the CNTs film with a three dimensional porous conductive framework structure as the working electrode (2 cm×1.5 cm), a Pt foil as the counter electrode, and an Ag/AgCl

electrode as the reference electrode. The employed electrolyte was prepared by dissolving manganese acetate tetrahydrate (0.1 M) and sodium sulfate anhydrous (0.1 M) into 500 mL deionized water. Then, the free-standing CNTs@MnO_2 film was obtained at 0.8 V for a duration of 200 s, and then dried at 60 °C for 1 h.

Preparation of GaIn-Ni semi liquid metal:

Typicaly, 0.8 g Nickel (Ni) nanoparticles (purchased from Aladdin Co.. Ltd) with a diameter of 20~100 nm were shear mixed with 7.2 g pristine GaIn liquid eutectic alloy, which is comprised of 76 wt% Ga and 24 wt% (purchased from Northeast Non-ferrous Metals Market Co., Ltd.), with a mortar and a glass rod at room temperature. After sufficient mechanical stirring for 1 h, the GaIn-Ni semi liquid metal was obtained.

Synthesis of zinc trifluoromethanesulfonate-manganese sulfate/polyacrylamide solid polymer electrolyte:

First, 3.0 g acryl amide (AM), $Zn(CF_3SO_3)_2$ (21.8 g), and $MnSO_4 \cdot H_2O$ (0.51 g) were dissolved in deionized water (30 mL). Then, 0.02 g N, N'-methylenebis(acrylamide) being cross-linker, and 0.03 mL N, N, N', N'-Tetramethylethylenediamine for accelerant were soluble in the above solution. Finally, 0.007g initiator (ammonium persulfate) was put in with rapid stirring for 1 h to obtain the solid polymer electrolyte (2 M $Zn(CF_3SO_3)_2$-0.1 M $MnSO_4$/PAM).

Fabrication of ZHMSCs based on MXene/BC@PPy hybrid film and CNTs@MnO_2 hybrid film electrodes:

First, interdigital circuit pattern designed via the AutoCAD software was upload to a commercial laser cutting machine. The laser power was set to 30% (1.5 W) and the speed was set to 150 mms^{-1}. Before the laser-cutting process, a 80 nm Au layer was magnetron sputtered on the surface of the MXene/PPy@BC hybrid films as a current collector. Then, with the aid of the laser cutting, the interdigital capacitor-type electrode and battery-type electrode with designed dimensions based on the MXene/PPy@BC hybrid film and CNTs@MnO_2 film were fabricated, respectively. Then, the obtained interdigital electrodes were affixed onto a flexible PET film supporter (3700 MPa, 0.1 mm in thickness) with double faced adhesive tape. After coating the obtained 2 M $Zn(CF_3SO_3)_2$-0.1 M $MnSO_4$/PAM gel on the electrodes as the top solid electrolyte for ion migration, the aqueous Zn-ions hybrid MSCs (ZHMSCs) were obtained. After that, the obtained ZHMSCs were deployed in the "islands" region and interconnected by screen-printed GaIn-Ni based circuits "bridge" on bottom elastic silicone film carrier with a mechanically deformable structure (Ecoflex 00-30, mix ratio is 1A:1B by weight). Then, a top silicone encapsulation layer with the same structure was overlaid on the bottom silicone substrate with semi-cured silicone serving as glue. After fully curing of the semi-cured silicone with heat treatment on a heating plate at 40 °C for 30 minutes, a flexible and stretchable ZHMSCA is finally fabricated.

Electrochemical measurements:

CV, GCD, and EIS (open circuit voltage with ±10 mV amplitude) measurements were carried out via an electrochemical workstation (CHI 660E, Chenhua). The areal capacitance (Cs, mF cm^{-2}) and energy density (Ws, uWh cm^{-2}) were calculated based on the GCD curves according to the subsequent equations:

$$C = Q/\Delta E = I\Delta t/\Delta E \quad (1)$$

$$Cs = C/S = I\Delta t/S\Delta E \quad (2)$$

$$Ws = 0.5C(\Delta E)^2/3600s \quad (3)$$

Herein, C, Q, I, Δt represent the total capacitance, the total charge, the discharge current, and the discharge time, respectively. ΔE is the potential window during the discharge process after IR drop, and S is the total area of the positive and the negative electrodes.

The ion diffusion coefficients were calculated according to the following equation:

$$D_{Zn^{2+}} = 0.5\left[\frac{RT}{n^2F^2AC\sigma}\right]^2 \quad (4)$$

where R is the gas constant (8.314 J K^{-1} mol^{-1}), T is the room temperature (298.15 K), A is the surface area of the electrode (0.3 cm^2), n is the number of electrons transferred (2), F is the Faraday constant (96500 C mol^{-1}), C is the concentration of Zn^{2+} (2×10^{-3} mol cm^{-3}), and σ is the slope of the plot of Z, against $\omega^{-1/2}$ based on $Z' = Rs + Rct + \sigma\omega^{-1/2}$.

Material Characterizations:

The microstructure and phase composition of the samples were revealed by Field-emission scanning electron microscopy (FE-SEM, S-4800, Hitachi, Japan), Transmission electron microscopy (TEM, JEM-2100, JEOL, Japan), and X-ray powder diffraction (XRD Bruker D8-ADVANCE) with an 18 kW advanced X-ray diffrac-tometer with Cu K_α radiation (λ=1.54056Å). X-ray photoelectron spectroscopy (XPS) was conducted with a Mg K_α achromatic X-ray source. Sheet resistance was measured by a standard four-point probe method (RST-9, Four-Probe Tech.).

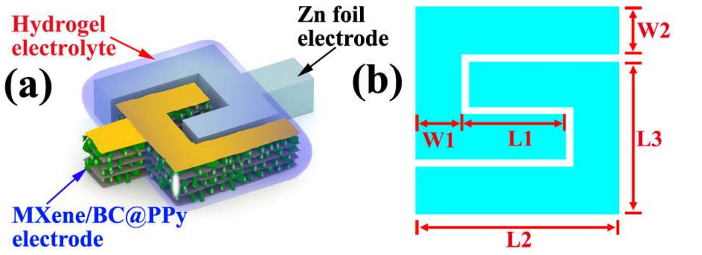

	Dimensions
L1	0.355 cm
L2	0.659 cm
L3	0.515 cm
W1	0.150 cm
W2	0.141 cm

Figure S1. Schematic diagram of the employed two-electrode electrochemical test system vs Zn foil electrode in a planar electrode configuration.

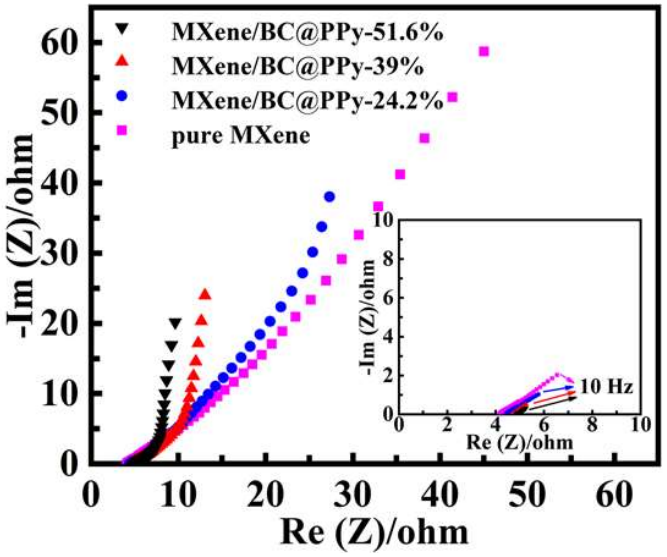

Figure S2. Comparison of the Nyquist plots of the MXene/BC@PPy hybrid electrodes with different mass percentage of BC@PPy nanofirbers (The inset is the enlarged plots at high frequency).

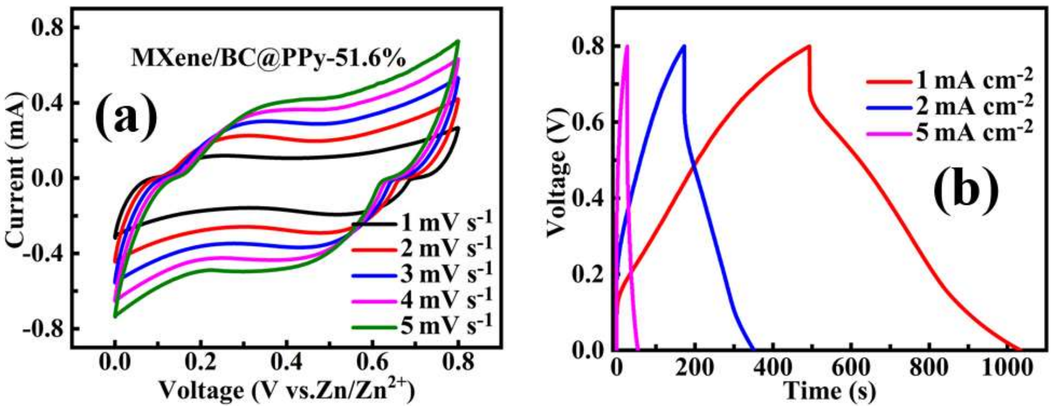

Figure S3. a) The CV curves and b) GCD curves of the MXene/BC@PPy-51.6% hybrid film electrode.

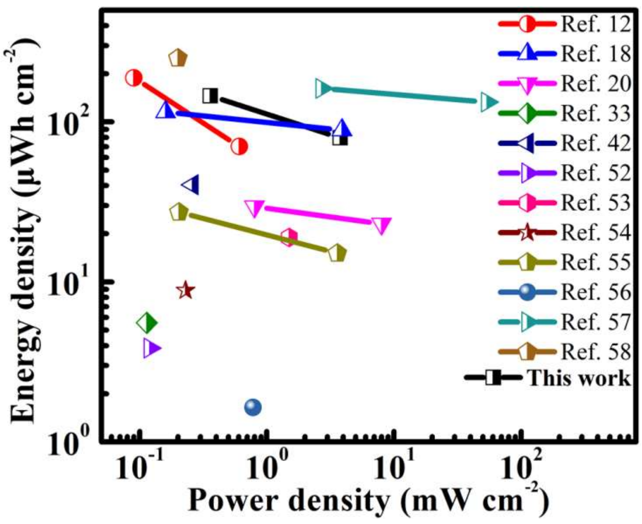

Figure S4. Ragone plot showing energy and power densities of the proposed ZHMSCs against state-of-the-art MXene-based MSCs, ZHMSCs based on carbon-based capacitor-type electrode, and ZMBs.
